# Methylation of Migraine-Related Genes in Different Tissues of the Rat

**DOI:** 10.1371/journal.pone.0087616

**Published:** 2014-03-07

**Authors:** Sieneke Labruijere, Lisette Stolk, Michael Verbiest, René de Vries, Ingrid M. Garrelds, Paul H. C. Eilers, A. H. Jan Danser, André G. Uitterlinden, Antoinette MaassenVanDenBrink

**Affiliations:** 1 Dept. of Internal Medicine, Div. of Pharmacology, Erasmus Medical Center, Rotterdam, The Netherlands; 2 Dept. of Internal Medicine, Genetics Laboratory, Erasmus Medical Center, Rotterdam, The Netherlands; 3 Dept. of Biostatistics, Erasmus Medical Center, Rotterdam, The Netherlands; Massachusetts General Hospital, United States of America

## Abstract

17ß-Estradiol, an epigenetic modulator, is involved in the increased prevalence of migraine in women. Together with the prophylactic efficacy of valproate, which influences DNA methylation and histone modification, this points to the involvement of epigenetic mechanisms. Epigenetic studies are often performed on leukocytes, but it is unclear to what extent methylation is similar in other tissues. Therefore, we investigated methylation of migraine-related genes that might be epigenetically regulated (CGRP-ergic pathway, estrogen receptors, endothelial NOS, as well as MTHFR) in different migraine-related tissues and compared this to methylation in rat as well as human leukocytes. Further, we studied whether 17ß-estradiol has a prominent role in methylation of these genes. Female rats (n = 35) were ovariectomized or sham-operated and treated with 17β-estradiol or placebo. DNA was isolated and methylation was assessed through bisulphite treatment and mass spectrometry. Human methylation data were obtained using the Illumina 450k genome-wide methylation array in 395 female subjects from a population-based cohort study. We showed that methylation of the *Crcp*, *Calcrl*, *Esr1* and *Nos3* genes is tissue-specific and that methylation in leukocytes was not correlated to that in other tissues. Interestingly, the interindividual variation in methylation differed considerably between genes and tissues. Furthermore we showed that methylation in human leukocytes was similar to that in rat leukocytes in our genes of interest, suggesting that rat may be a good model to study human DNA methylation in tissues that are difficult to obtain. In none of the genes a significant effect of estradiol treatment was observed.

## Introduction

Migraine is a neurovascular disorder affecting 8–17% of the population [1], [2]. Little is known about the cause of migraine and the prophylactic medication that is used to prevent migraine is only effective in half of the patients [3]. Family studies show a heritability of ∼40% [4], [5] and a monogenetic inheritance pattern has only been identified for familial hemiplegic migraine. Hence, there may be a major contribution of environmental (including hormonal) influences, possibly via epigenetic mechanisms. The methylene tetrahydrofolate reductase gene (*MTHFR*), encoding an important protein of the DNA methylation cycle, has been suggested to be involved in migraine [6], [7], although genome-wide association studies did not confirm this. However, they pointed to other genes involved in epigenetic mechanisms [8]–[10]. Moreover, the migraine prophylactic valproate, inhibits histone deacetylation as well as DNA methylation [11]. Finally, 17β-estradiol, which seems to be responsible for the 2–3 times higher prevalence of migraine in women compared to men, may exert at least part of its effects via epigenetic mechanisms [12], [13]. Thus, DNA methylation may be involved in migraine pathophysiology.

DNA methylation occurs at cytosines of CpG dinucleotides, often localized in CpG rich regions called CpG islands [14]. CpG island methylation is changed in different types of cancer, during aging, and probably also during the menstrual cycle [15]–[18]. Environmental factors that might be responsible for these epigenetic mechanisms include female hormones and nutrition [19], [20].

DNA methylation can differ greatly between tissues, so ideally it should be studied in the tissue of interest. For complex brain diseases like migraine, this is technically impossible in humans. Yet, blood can be derived easily, and thus if the DNA methylation pattern of leukocytes is correlated to that of other tissues, this would allow conclusions from methylation studies in leukocytes.

The aim of our study was to compare the methylation of genes that are probably involved in the generation of a migraine attack and might be epigenetically regulated [21], [22], as well as to examine the role of 17ß-estradiol in the methylation of our genes of interest. We focussed on the CGRP-ergic system because of its prominent role in migraine [23] We investigated DNA methylation in the Calcitonin related peptide alpha *(Calca)*, receptor activity-modifying protein 1 *(Ramp1)*, calcitonin receptor component protein *(Crcp)*, calcitonin receptor-like receptor *(Calcrl)*, upstream stimulating factor 2 *(Usf2)*, Estrogen receptor 1 *(Esr1)*, G-protein coupled estrogen receptor 1 *(Gper)*, nitric oxide synthase 3 *(Nos3)*) and *Mthfr* genes in several tissues relevant to the pathophysiology of migraine (dura mater, trigeminal ganglion and trigeminal caudal nucleus). We investigated methylation in leukocytes and aorta as a peripheral control. To investigate whether DNA methylation in rats may be representative of that in humans, we also studied DNA methylation in leukocytes obtained from healthy human females.

## Materials and Methods

### Ethics statement

The animal experiments were performed in our laboratory with permission of the ethics committee of the Erasmus Medical Center in Rotterdam, The Netherlands (Permit Number: EMC2345(127-11-02)). All surgeries were performed under sodium pentobarbital anesthesia and all effort was made to minimize suffering.

The human blood samples were obtained from the Rotterdam Study [24], which has been approved by the institutional review board (Medical Ethics Committee) of the Erasmus Medical Center and by the review board of The Netherlands Ministry of Health, Welfare and Sports. All subjects provided written informed consent.

### Animals

Female Sprague Dawley rats (Harlan Netherlands, Horst, The Netherlands) (N = 11–12 per group, weight at the start of the study ∼250 g) (See Table S1 in File S1) were kept at room temperature (22°C) at a 12/12 hours dark/light cycle with unlimited access to food and water in their home cages. The animals were anesthetized (50 mg/kg) and ovariectomized or sham-treated on day 1 of the study. After 7 days, a pellet releasing placebo or 17β-estradiol (21-day release pellet, 12 µg/day, Innovative Research, USA) was implanted subcutaneously in the neck. Blood samples were collected on day 1, 7 and 21 for CGRP and hormone measurements (See Table S2 in File S1). On day 21, the animals were sacrificed via an overdose of sodium pentobarbital (200 mg/kg). A leukocyte differentiation count was performed to verify whether the proportion of different types of leukocytes was the same for all animals. 0.5 ml whole blood, dura mater, trigeminal ganglia, caudal nuclei and a 5-mm segment of thoracic aorta were snap frozen in liquid nitrogen and stored at −80°C for DNA isolation. A vaginal smear was taken at all three time points to establish the phase of the estrous cycle according to proportions of epithelial cells, cornified cells and leukocytes present in the smear. Because an epigenetic study as in the current experiments has not been performed previously, we based the number of animals on our previous results, showing increased vascular endogenous CGRP responses after treatment with 17β-estradiol [25] and explored whether differences in DNA methylation caused by 17β-estradiol can be demonstrated in this model.

### DNA methylation measurements

DNA of leukocytes, thoracic aorta, dura mater, trigeminal ganglia and caudal nuclei from all animals was isolated (DNeasy, Qiagen, Germantown, MD, USA) and quantified (Nanodrop, Thermo Scientific, Wilmington, DE, USA).

CpG islands of the genes of interest were determined using UCSC (http://genome.ucsc.edu/) and Ensemble genome browsers (http://www.ensembl.org). Primers were designed for each CpG island located in the promoter region of the genes of interest with the EpiDESIGNER primer design software (Sequenom, San Diego, CA, USA, Table 1). When the CpG island could not be covered in total, multiple primer sets were designed to cover the largest possible part of the CpG Island. With the BiSearch web server [26] primer sets were checked for having only one product.

**Table 1:** Primers and conditions for PCR on CpG island located in the promoter region of genes of interest.

Amplicon	Primer Sequence	Amplicon size (bp)	Number of analyzed CpG's
*Calca-part 1*	Forward-TTTAAATGGTGTTATTTTGTTAGATGTT Reverse-TAAACAAAAACCTCAAAACTCACCT	282	7
*Calca-part 2*	Forward-GTAATTGTGGTTGTTGGTTTTTGTT Reverse-CACCAAATAAACCCTAAAATTCCTA	405	7
*Ramp1*	Forward-GGGGGTTATGGTAAGTAGAGTTT Reverse-TTACAAAACAAACCCCAAAATAACT	294	17
*Crcp*	Forward-TTAGTAGTGGGTTTAGGAAGAGAGTG Reverse-CTAAAAAACAAAATTCTAAATACACAAAAC	290	11
*Calcrl*	Forward-GGGTTTTGTTGTTTGGATTTTA Reverse-TCCAAAAACTTACCTTATAACCTATTCA	244	2
*Usf2*	Forward-GGTAGTAGTGTTGATTTTGGTGGG Reverse-TCACCTAACCTCCATTACTCTCTATAC	411	15
*Esr1-part 1*	Forward-GGTAGTAGGGTATTTGGTGGTTATG Reverse-CAACTCAAAATACCCATAAAAAAAA	321	9
*Esr1-part 2*	Forward-TTTTTTGATTTTTTAGAAGGGTGG Reverse-TAATCTAAAAACTTTCCCCCAACTC	293	13
*Gper1-part 1*	Forward-GAAGATTATTTTTAGGGTTTTTTGTTTG Reverse-ACACCTTCATATCCCTCTTCCTACAA	397	7
*Gper1-part 2*	Forward-TTGTTGTATATGTTGATTTGTAGGAAGA Reverse-CCCAAACCTATACTTCATCAACCTAA	152	2
*Nos3*	Forward-TTTTTGTAAAGAAAAATTTTGGGTG Reverse-CACAATAAAAACTACCCCTAAACCT	404	11
*Mthfr*	Forward-GGGGTGATAGTTATTATAAGTTTATAGGTT Reverse-TTAACTAACTTCCCAAAAAACCTCC	244	12

A 10mer (aggaagagag) sequence was added to the forward primers and a T7 sequence (cagtaatacgactcactatagggagaaggct) to the reverse primers. The primers were obtained from Invitrogen (Life Technologies Corporation, Carlsbad, CA, USA).

500 ng DNA of all tissue samples was treated with bisulfite (EZ-96 DNA-methylation kit (Shallow), Zymo Research, Irvine, CA, USA) for 16 hours to convert all non-methylated cytosines into uracil nucleotides. After bisulfite conversion PCR, reverse transcription and uracil specific cleavage was performed. For quantitative DNA methylation measurements the MassARRAY EpiTYPEr was used (Sequenom, San Diego, CA, USA). 2.5 µg of low methylated and 2.5 µg high methylated genomic rat DNA (EpigenDx, Hopkinton, MA, USA) were mixed into 0%, 25%, 50%, 75% and 100% methylated DNA as a control and also treated with bisulfite. To check the accuracy of the methylation measurements, methylation of a standard curve, of which the % of methylation is known (0–100%, increasing in steps of 10%), was measured for all amplicons (See Figure S1 in File S1). After the validation steps, a PCR was performed in triplo for all amplicons on the bisulfite-treated samples. Percentage methylation for each CpG in all genes and tissues was calculated with Sequenom EpiTYPER software (MassARRAY EpiTYPER Analyzer software v1.0, build1.0.6.88 Sequenom, Inc, San Diego, USA). Samples were excluded when the standard deviation of triplos was larger than 10%.

### Genome-wide methylation study in humans

DNA methylation profiles from whole blood were assessed in a subset of 395 healthy women (age ≥45) from the Rotterdam Study-III, a population-based cohort study in the Netherlands. The design and rationale of the study has been published previously [24]. Samples were excluded when showing a low detection rate (<99%), incomplete bisulfite conversion, or gender swaps. Probes with a detection p-value >0.01 in >1% samples, were filtered out. β-values for the two assay-types on the array were corrected with SWAN (Subset-Within-Array-Normalization). Mean and standard deviation for the probes in the equivalent human genomic regions were calculated for 395 samples that passed quality control filters.

### Data analysis

**Figure 1:**
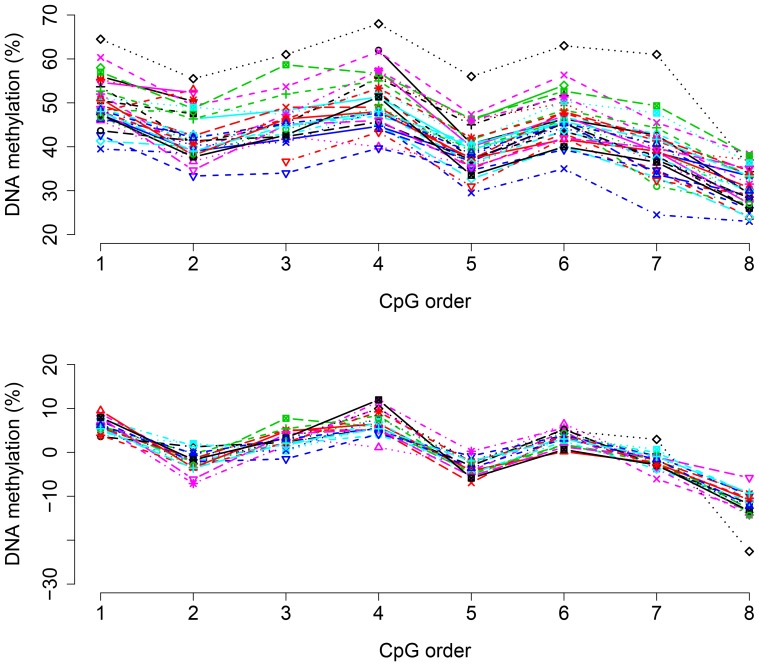
Methylation of the *Crcp* gene in the trigeminal ganglion. Methylation of each studied CpG of the promoter region of the *Crcp* gene is shown without correction for rat levels (upper panel) and with correction for rat levels (lower panel).

Calculations of differences in methylation and correlation between tissues were performed using SPSS software. The linear mixed model was used to estimate the size and standard errors of the effects of tissue and treatment. The model contains random effects for the individual rats, to correct for the strong correlations between CpGs within a gene. Typically, when one CpG shows a low (or high) level of methylation for a chosen rat, all the other CpGs show a low (or high) level as well. A strong example is shown in Fig. 1, for the *Crcp* gene in the trigeminal ganglion. The profiles for different rats run more or less in parallel, but at quite different levels. Subtracting the mean of all CpGs per rat removes the differences almost completely. The computations were done with the R system [27], version 2.15.2, using the function lmer in the library lme4.

## Results

### DNA methylation measurements

**Figure 2:**
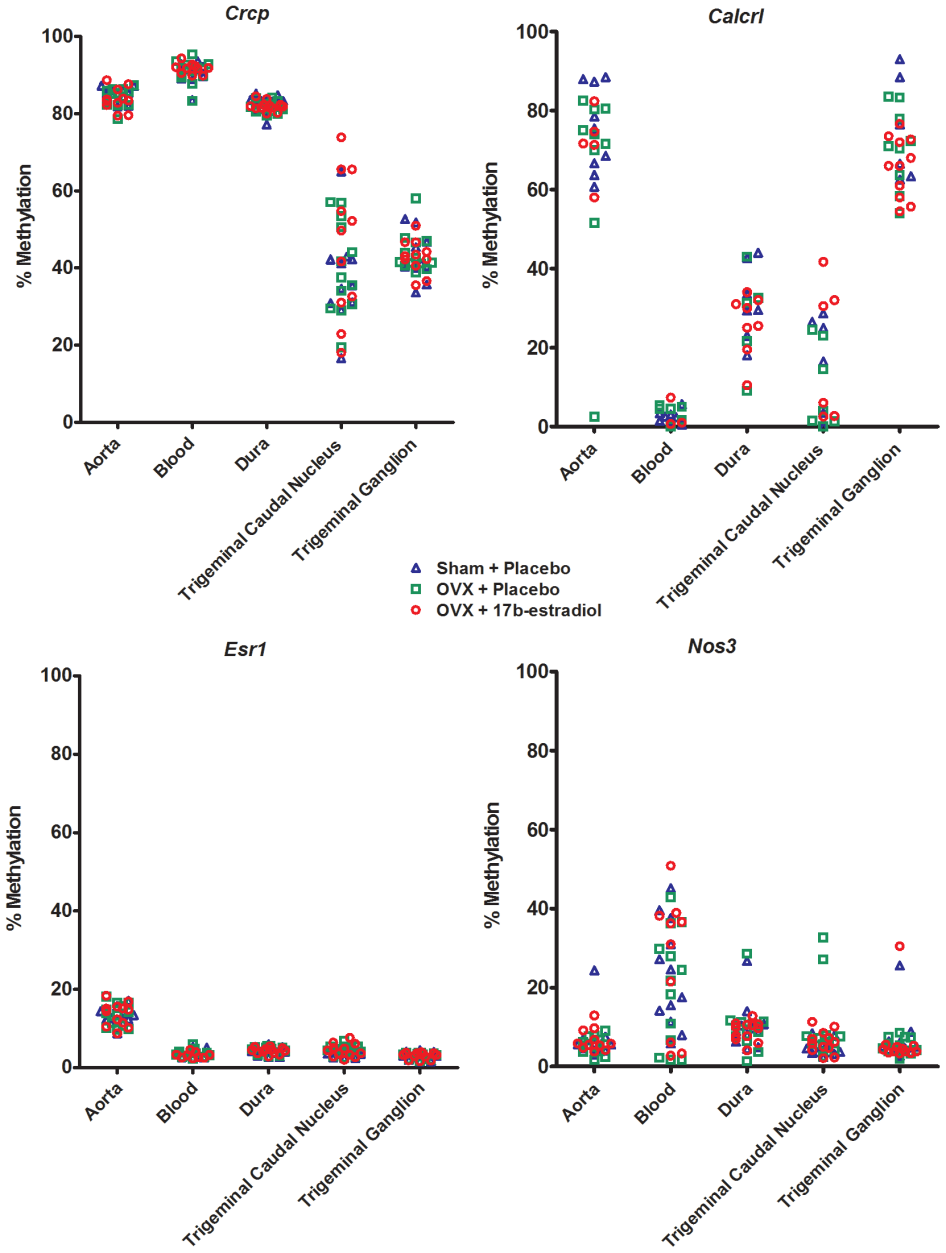
DNA methylation of *Crcp*, *Calcrl*, *Esr1* and *Nos3* in different tissues. Mean methylation of the promoter region of the *Crcp* (upper left panel), *Calcrl* (upper right panel), *Esr1* (lower left panel) and *Nos3* (lower right panel) genes is shown for the different tissues studied. Each symbol represents one animal and the different colors and symbols represent the treatment of the animals. Red circle: OVX+17β-estradiol; Green square: OVX+placebo; Blue triangle: sham+placebo.

Because all CpGs per CpG island that we studied were strongly correlated, we calculated the mean methylation of a CpG island and used these values for further analysis (see paragraph 2.4, Statistics). DNA methylation below 5% was observed for the *Calca, Ramp1, Usf2 and Mthfr* genes in all tissues (Table 2). DNA methylation higher than 90%, was observed for the *Gper* gene in all tissues (Table 2). DNA methylation differed substantially between tissues for the *Nos3*, *Esr1*, *Crcp* and *Calcrl* genes. *Nos3* showed a two times significantly higher DNA methylation in leukocytes than in the other tissues. Methylation of the *Crcp* gene was high in aorta, leukocytes and trigeminal caudal nucleus, and significantly lower in dura mater and trigeminal ganglion. The *Calcrl* gene showed low methylation in leukocytes, intermediate methylation in dura mater and trigeminal caudal nucleus and high methylation in aorta and trigeminal ganglion. DNA methylation of *Esr1* was significantly higher in the aorta than in other tissues (Fig. 2 and Table 2). Remarkable is that the variation in DNA methylation is small in some tissues and large in others. This is not a tissue-specific pattern, but it varies between tissues for the *Nos3*, *Esr1*, *Crcp* and *Calcrl* genes, as illustrated by the differences in standard deviation (Table 2).

**Table 2:** Mean methylation of CpG islands of candidate genes in aorta, leukocytes, dura mater, trigeminal caudal nucleus and trigeminal ganglion.

	Aorta	Leukocytes	Dura	Trigeminal Caudal Nucleus	Trigeminal Ganglion
	Mean ± SD (%)	Mean ± SD (%)	Mean ± SD (%)	Mean ± SD (%)	Mean ± SD (%)
*Calca* *	2.3±0.6	4.2±1.6	2.4±0.7	2.8±1.0	2.5±1.1
*Ramp1* *	2.3±0.5	2.8±0.8	2.3±0.5	3.2±0.7	2.5±0.6
*Crcp* *	84.3±2.4	91.0±2.5	82.1±1.6	41.1±14.1	43.2±5.1
*Calcrl* *	70.6±17.6	2.9±2.2	28.2±9.6	15.0±13.5	69.6±10.3
*Usf2*	1.5±0.3	1.7±0.6	1.5±0.3	1.5±0.3	1.6±0.3
*Esr1* *	13.4±2.6	3.4±0.9	4.3±0.9	4.1±1.3	3.0±0.8
*Gper* *	93.7±0.8	93.4±1.7	92.6±0.7	90.2±2.2	92.9±0.8
*Nos3* *	6.4±3.9	23.0±14.6	9.7±5.1	7.1±6.0	6.1±5.6
*Mthfr*	2.0±0.4	2.0±0.6	1.9±0.5	2.2±0.6	1.9±0.4

*) Significant differences between tissue means are present within the respective gene. P-value <0.0001.

Estradiol treatment did not induce any statistically significant differences in DNA methylation in any of the examined genes in any investigated tissue (Fig. 2). Therefore, the data of the different treatment groups were pooled for further statistical analyses, where treatment effect was not included.

The DNA methylation of leukocytes and that of the other tissues were compared to each other for all genes. No correlation was seen between DNA methylation in the leukocyte samples and samples from other tissues (Table 3). A leukocyte differentiation analysis was performed, but no influence was found of leukocyte composition on methylation of blood samples for any of the genes (data not shown).

**Table 3:** Relationship of methylation of leukocytes with other tissues.

	Methylation of leukocytes compared to other tissues (Pearsons r^1^)			
	Aorta	Dura	Trigeminal Caudal Nucleus	Trigeminal Ganglion
***Calca***	−0.23	−0.33	−0.38	−0.01
***Ramp1***	0.26	0.64	0.40	0.40
***Crcp***	0.04	0.33	0.09	0.17
***Calcrl***	0.08	0.29	0.52	−0.37
***Usf2***	0.07	0.50	0.07	−0.06
***Esr1***	0.43	0.53	0.18	0.58
***Gper***	0.01	0.02	0.17	0.07
***Nos3***	0.02	0.18	−0.36	−0.38
***Mthfr***	0.33	−0.09	−0.02	0.13

1 ) The relevance of the correlations can best be appreciated from a prediction perspective. Assume that we are interested in estimating the mean amount of methylation of some gene in some tissue. If we have no specific information, the best we can do is take the observed mean in our sample. The uncertainty is quantified by the observed standard deviation (SD). If a linear relationship with the mean amount of methylation in blood exists, a prediction model could be derived. Using basic statistical theory, one can show that the uncertainty is reduced to c * SD, where c follows form the formula c^2^ = 1−r^2^, if r is the correlation coefficient. To get c = 0.5, thus halving the uncertainty, r has to be as high as 0.87. Conversely, when r = 0.5, c = 0.87 and thus only a 13% reduction is obtained. For reliable prediction really high correlations (e.g. r = 0.97 for c = 0.25) are needed.

### Concordance of DNA methylation in human and rat leukocytes

**Figure 3:**
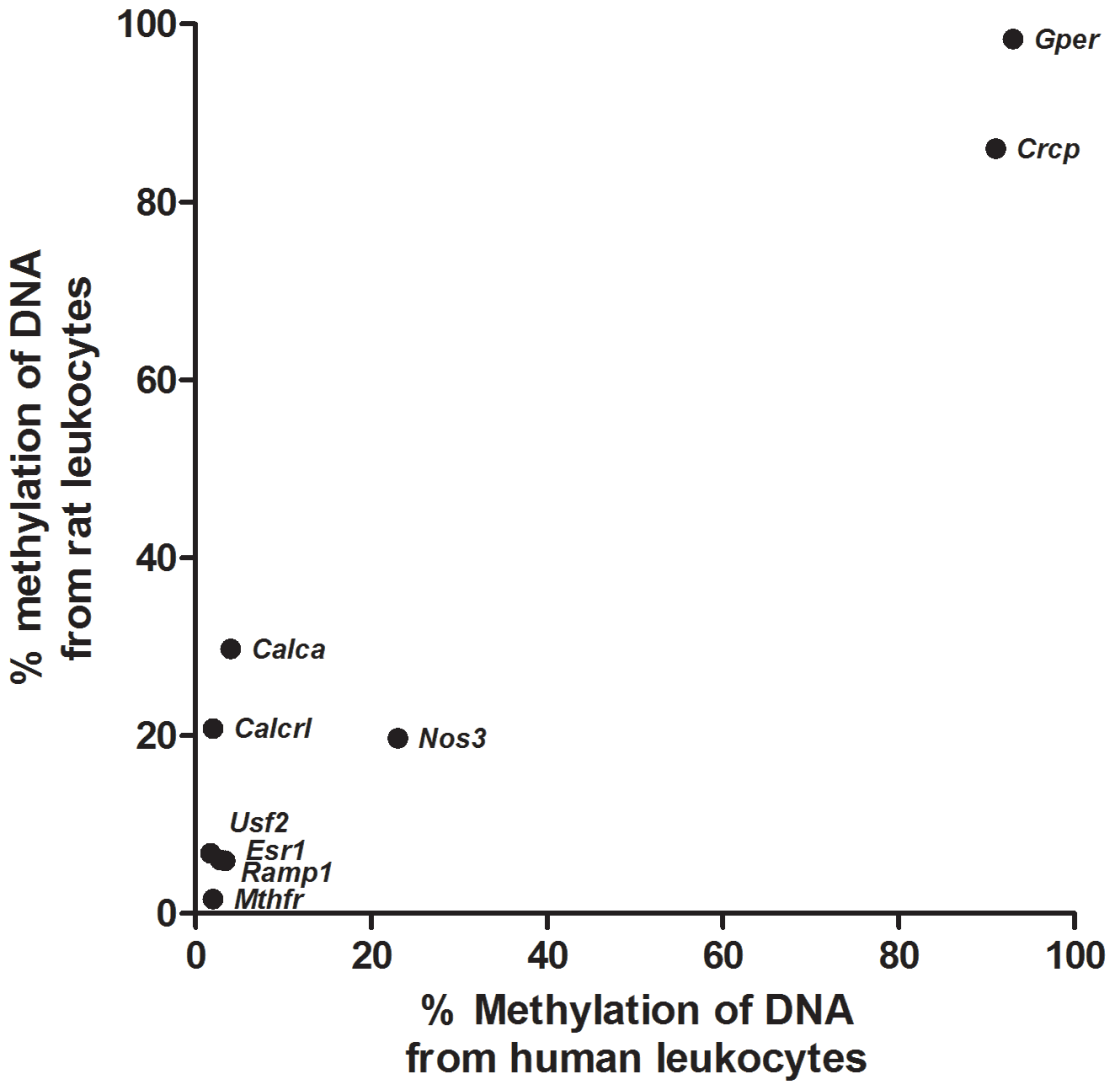
Rat leukocyte DNA methylation compared to human leukocyte DNA methylation. The methylation of DNA from rat leukocytes is compared to methylation of DNA from human leukocytes for different migraine-related genes. Our genes of interest that are high methylated in rat leukocytes, are also high methylated in human leukocytes and the genes that are low methylated in rat leukocytes are also low methylated in human leukocytes.

Human female DNA methylation data of our genes of interest were obtained from a genome wide methylation array. Human DNA methylation data of exactly the same genomic region of rat DNA was available for the *CALCA*, *CALCRL*, *USF2*, *ESR1*, *GPER* and *MTHFR* genes. For the other genes, *RAMP1, CRCP and NOS3*, the region studied in rat was very close (<100 bp) to the regions studied in human. We observed comparable values of DNA methylation in our genes of interest in human leukocytes as in rat leukocytes; the two genes with a high percentage of DNA methylation in the rat had also a high percentage of DNA methylation in the human samples, while the seven genes with a low percentage of DNA methylation were also concordant between the rat and human samples (Table 4, Fig. 3).

**Table 4:** Methylation of amplicons of rat genes compared to homologues regions of the human genome.

		*% DNA methylation Rat*	*% DNA methylation human*	*Location of rat amplicons in human genome (Build 37)*	*Location Illumina probes (Build 37)*	*Accession Number Rat*	*Accession Number Human*
***High DNA methylation***	***Crcp***	91.0±2.5	86.0±5.8	7:65587384–7:65587475	7:65583242–7:65604635	NM_053670.3	NM_014478.4
	***Gper1***	93.4±1.7	98.3±0.8	7:1131643–7:1132074	7:1132036	NM_133573.1	NM_001505.2
***Low DNA methylation***	***Calca***	4.2±1.6	30.0±3.8	11:14993934–11:14994000	11:14993929–11:14293977	NM_017338.2	NM_001741.2
	***Calcrl***	2.9±2.2	20.8±5.7	2:188312541	2:188312833	NM_012717.1	NM_005795.5
	***Esr1***	3.4±0.9	6.1±2.3	6:152129135–6:152129504	6:152129388–6:152129400	NM_012689.1	NM_000125.3
	***Mthfr***	2.0±0.6	1.8±1.0	1:11866155–1:11866365	1:11866155–1:11866236	NM_005957.4	XM_001074061.4
	***Nos3***	23±14.6	19.7±4.3	7:150710828–7:10571127	7:150710730–7:150711138	NM_021838.2	NM_000603.4
	***Ramp1***	2.8±0.8	6.1±2.3	2:238768163–2:238768240	2:238768104–2:238828641	NM_031645.1	NM_005855.2
	***Usf2***	1.7±0.6	6.8±2.3	19:345760455–19:35760666	19:35760554	NM_031139.1	NM_003367.2

## Discussion

### DNA methylation and gene expression across tissues

DNA methylation and histone modifications, making the DNA accessible or locked for transcriptional regulation, are responsible for physiological processes like stem cell differentiation. Therefore, CpG island methylation may be similar between tissues but can also differ substantially. DNA methylation of the *Calca*, *Ramp1*, *Usf2* and *Mthfr* genes was low in all tissues. Not taking into account other regulatory mechanisms, expression of these proteins would thus be expected in all investigated tissues. Notwithstanding the fact that tissue-specific information is not always available, all four genes are indeed expressed in several brain and cardiovascular tissues [28]–[33]. The expression in human leukocytes [34]–[37] suggests the expression in rat leukocytes as well.

In contrast, DNA methylation of the CpG island of the *Gper* gene was 90–94% in all investigated tissues, suggesting that expression of this gene might be low or absent. However, *Gper* mRNA is present in cells of the aorta [38] and in the central and peripheral nervous system [39] But the quantitative extent of this expression is unknown. Further, regulation of protein expression might even occur at high percentages of DNA methylation. Obviously, other regulatory mechanisms, such as histone modifications or non-epigenetic mechanisms like transcription factor binding to specific regions on the DNA, may also have an additional effect on protein expression. Hence, DNA methylation is not necessarily related to protein expression for every gene [40], [41].

Our results show that the variation in methylation differs between tissues in the *Crcp*, *Calcrl*, *Esr1* and *Nos3* genes, which are expressed in several brain and vascular tissues [32], [42]. *ESR1* and *NOS3* are also expressed in human leukocytes [43], [44] Large variations could result from contamination with other tissues, but variations would then be expected to be small in tissues that are a distinct anatomical entity, such as aorta and trigeminal ganglion. Furthermore, outliers potentially caused by contaminated tissue, should then be the same in all examined genes, which was not the case. It would be tempting to quantitatively relate differences in DNA methylation to differences in gene expression and subsequent translation into proteins in the different tissues. However, such a quantification between tissues, which is clearly beyond the scope of our study, would be hampered by the different characteristics of the tissues studied, with the consequent lack of a validated internal standard between tissues. Taken together, the substantial variations in DNA methylation between tissues for *Crcp*, *Calcrl*, *Esr1* and *Nos3* genes in some of the tissues (Fig. 2), suggests a regulatory role for DNA methylation in the expression of these genes in the respective tissues.

### DNA methylation in leukocytes versus other tissues

DNA methylation is regularly examined in leukocytes with the aim of discovering biomarkers for several diseases [45], [46] because of the difficulty of obtaining affected tissues. For example, leukocyte DNA methylation is correlated to different types of cancer [47]–[49]. In our study, there was no correlation between DNA methylation of the candidate genes in leukocytes and that in the other tissues. Thus, it can be concluded that leukocytes are not representative for changes in DNA methylation in aorta, dura mater, trigeminal caudal nucleus and trigeminal ganglion for the migraine-related genes we investigated (Table 2).

### Is the rat a valid model for human DNA methylation?

We studied whether DNA methylation of human leukocytes in our genes of interest is similar to DNA methylation of rat leukocytes. Since DNA methylation in human and rat leukocytes was found to be concordant, we hypothesize that this might also be the case for other tissues. This finding, combined with the obvious limitations in obtaining human tissues, suggests that animal models are relevant to study DNA methylation changes in specific tissues.

### DNA methylation and environmental factors

DNA methylation may be influenced by environmental factors like nutrition [50] and maternal care [51]. Hormones like 17β-estradiol may also alter DNA methylation [52], [53]. Interestingly, female hormones probably play an important role in migraine as migraine is much more common in females than in males especially during the fertile part of their lives, and migraine attacks in females often start at the day before menstruation [54]. It is well known that estradiol affects epigenetic mechanisms [12], [13]. In addition, it potentiates the response to an important peptide in migraine pathophysiology, calcitonin gene-related peptide (CGRP) [24], [25], which is widely expressed in the central and peripheral nervous system [55]. We previously demonstrated that estrogen increases vascular sensitivity to CGRP and trigeminal innervation [25]. Estrogen can also modulate CGRP expression in the spinal portion of the trigeminal caudal nucleus and the cervical spinal cord [56], [57]. How estrogen causes these effects is still unknown, but a recent study showed that CGRP expression is epigenetically regulated [21]. Epigenetic mechanisms like DNA methylation may thus be involved in the differences in migraine prevalence between men and women.

When examining mean values of DNA methylation of the three different treatment groups (Fig. 2) some differences were observed between the groups, but these were not statistically significant. Because we are, to the best of our knowledge, the first to perform a study on DNA methylation of candidate genes in our field, we could not perform an adequate power calculation. We based our group sizes on our previous studies using the same animal model [25], where the same dose of and duration of treatment with 17β-estradiol as in the current study were adequate to induce changes in CGRP-ergic pathways. Furthermore, others have shown that DNA methylation patterns can change within hours or days [16], [58]. A study with a similar design as our current study showed that an environmental factor, bisphenol A, can increase methylation of the promoter region of the estrogen receptor [59], indicating that the methods we applied are suitable to detect changes in DNA methylation due to environmental factors.

The large variation in methylation in our investigated tissues is likely to have reduced the statistical power of our study when investigating the effects of 17ß-estradiol treatment. We observed a maximal difference of 8% methylation with a standard deviation of 18% between animals treated with 17β-estradiol and animals treated with placebo (data not shown). Based on a post-hoc power calculation, a group size of 318 animals would be needed to obtain sufficient statistical power to investigate these hormone effects. This is practically not feasible so another study setup, possibly an in vitro study, falling beyond the scope of the current study, is needed to investigate the effect of 17β-estradiol on DNA methylation. Thus, with our experiment, we cannot categorically exclude that the methylation of the genes that we have studied may be influenced by 17ß-estradiol.

### Relevance of CpG islands

Methylation of CpG islands in and close to promoter regions of genes can cause a decrease in transcription of those genes during development and in certain types of cancer, Therefore, the focus in DNA methylation studies, including our current study, is often on CpG islands [60], [61]. CpG islands in or close to the promoter region of a gene are only present in 60% of the genes. Also in our case, there are genes that were of potential interest because they are involved in CGRP and estrogen signalling, as for example estrogen receptor 2 (*Esr2*) or the transient receptor potential cation channel, subfamily V, member 1 (*Trpv1*), but that are devoid of CpG islands in the rat genome. Therefore, we did not investigate these genes in our study. Recently, it has been shown that also methylation changes in enhancer regions of the DNA [62], as well as the CpG island shores [63], may induce differences between subjects, providing another regulatory region in which DNA methylation plays a role in the regulation of transcription. The general relevance of these findings, however, needs to be confirmed in future studies.

### Overall conclusion

In conclusion, our results show that DNA methylation of the rat genes we studied is variable, tissue specific and cannot be extrapolated from leukocytes to other tissues. On the other hand, we observed a high degree of concordance between human and rat DNA methylation in leukocytes, suggesting that it is possible to study effects on DNA methylation in rat tissues that are difficult to obtain from humans, for example several types of brain tissues. The large variation of DNA methylation in the *Crcp*, *Calcrl*, *Esr1* and *Nos3* genes suggests that these genes are prone to changes in DNA methylation, but challenges drawing conclusions about the effect of 17β-estradiol.

## Supporting Information

File S1 **Figure S1.** Example of standard curve, showing the distribution of methylation of the known samples for the *Esr1* gene. **Table S1.** Body weight (g) and bodyweight changes (Δ, g) in rats after different treatments (n = 11–14). * p<0.05 compared to OVX placebo. Differences were calculated using one-way ANOVA. **Table S2.** Estradiol and CGRP concentrations measured at day 1, day 7 and day 21 and progesterone concentrations measured at day 21. * p<0.05 compared to both sham and OVX animals treated with placebo pellet. Differences were calculated using one-way ANOVA.
(DOCX)
