# Supplementary material for: Methylation of Migraine-Related Genes in Different Tissues of the Rat
Source: PLoS One. 2014 Mar 7;9(3):e87616. doi: 10.1371/journal.pone.0087616 (PMC3946422; doi:10.1371/journal.pone.0087616)
Supplement: File S1 — Figure S1. Example of standard curve, showing the distribution of methylation of the known samples for the Esr1 gene. Table S1. Body weight (g) and bodyweight changes (Δ, g) in rats after different treatments (n = 11–14). * p<0.05 compared to OVX placebo. Differences were calculated using one-way ANOVA. Table S2. Estradiol and CGRP concentrations measured at day 1, day 7 and day 21 and progesterone concentrations measured at day 21. * p<0.05 compared to both sham and OVX animals treated with placebo pellet. Differences were calculated using one-way ANOVA. (DOCX) [file pone.0087616.s001.docx]

Supplement

Results

Body weight, vaginal smears and estradiol, CGRP and progesterone concentrations

To establish that the ovariectomy (OVX) operation and pellet implantation were successful, estradiol concentrations were measured in blood plasma at day 1, day 7 (at the end of the implantation procedure) and day 21. At day 7 and 21 an increased estradiol concentration is seen in ovariectomized animals treated with estradiol pellets (See Table S2 in File S1). Furthermore, vaginal smears were examined to evaluate cyclic activity. Cyclic activity was reduced after OVX and increased after consequent treatment with estradiol pellets, compared to the sham-operated animals, which showed a normal cycle (results not shown). Body weights of the animals in the three different treatment groups are shown in Table 1 of the supplement. No differences in bodyweight were seen at day 1 and day 7. The increase in bodyweight at day 21 in the ovariectomized animals treated with placebo pellet was significantly larger than that in the ovariectomized animals that were treated with an estradiol pellet. Concentrations of CGRP, at day 1, 7 and 21, and progesterone, at day 21, were also measured and no significant difference was found for the different treatment groups (See Table S2 in File S1).

Tables

**Table S1.** Body weight (g) and bodyweight changes (Δ, g) in rats after different treatments (n=11-14). * p < 0.05 compared to OVX placebo. Differences were calculated using one-way ANOVA.

|  | **Sham-operated rats** | **Ovariectomized rats treated with:** | |
| --- | --- | --- | --- |
|  |  | **Placebo** | **17β-estradiol** |
| **Day 1** | 254±3 | 256±2 | 255±4 |
| **Day 7** | 253±4 | 259±2 | 255±5 |
| **Day 21** | 266±3 | 280±6 | 254±3 |
| **Δ Body Weight at day 21** | 11±3 | 25±7 | -1±4* |

**Table S2.** Estradiol and CGRP concentrations measured at day 1, day 7 and day 21 and progesterone concentrations measured at day 21. * p < 0.05 compared to both sham and OVX animals treated with placebo pellet. Differences were calculated using one-way ANOVA.

|  | | **Sham-operated rats treated with:** | **Ovariectomized rats treated with:** | |
| --- | --- | --- | --- | --- |
|  | | **Placebo pellet** | **Placebo pellet** | **17β-estradiol pellet** |
| **Estradiol** | Day 1 | 12±1 | 25±8 | 20±5 |
| **concentration** | Day 7 | 19±11 | 9±1 | 130±39* |
| **(pg/ml)** | Day 21 | 16±5 | 12±3 | 133±14* |
| **CGRP** | Day 1 | 69±4 | 74±4 | 73±4 |
| **concentration** | Day 7 | 77±4 | 61±7 | 74±4 |
| **(pg/ml)** | Day21 | 67±4 | 68±3 | 69±4 |
| **Progesterone** | Day 21# | 12±3 | 10±2 | 16±5 |
| **concentration** |  |  |  |  |
| **(pg/ml)** |  |  |  |  |

**#)** Progesterone could not be measured at day 1 and 7 because of the limited availability of blood.

Figures

**Figure S1**

**Figure S1**. Example of standard curve, showing the distribution of methylation of the known samples for the *Esr1* gene.
